# Current Perspectives on Chitinolytic Enzymes and Their Agro-Industrial Applications

**DOI:** 10.3390/biology10121319

**Published:** 2021-12-12

**Authors:** Vikram Poria, Anuj Rana, Arti Kumari, Jasneet Grewal, Kumar Pranaw, Surender Singh

**Affiliations:** 1 Department of Microbiology, Central University of Haryana, Mahendargarh 123031, India; vikramporia@gmail.com (V.P.); arti12c@gmail.com (A.K.); 2 Department of Microbiology (COBS & H), CCS Haryana Agricultural University, Hisar 125004, India; anuj23rana@gmail.com; 3 Department of Environmental Microbiology and Biotechnology, Institute of Microbiology, Faculty of Biology, University of Warsaw, Miecznikowa, 102-096 Warsaw, Poland; g.jasneet@gmail.com (J.G.); kpranaw@gmail.com (K.P.)

**Keywords:** chitinolytic enzymes, microbial chitinases, chitooligosaccharides, fermentation, biocontrol

## Abstract

**Simple Summary:**

Chitin is a polysaccharide that forms the outer layer of many organisms, and it is widely used in industry. Chitinases are enzymes that can break down chitin into monomeric molecules and are used in the agro-industrial sectors. Because chitin is the key structural component of marine (mollusks, crustaceans, and marine invertebrates) and other species (algae, fungi, and insects), chitinases can be employed in the marine waste management and biocontrol of pathogenic fungi and harmful insects. Chitinase also has uses in the food industry, cosmetics, medicine, waste management, crop protection, and the production of single-cell proteins, among others. This study includes detailed information on the characterization, sources, and uses of chitinases in several areas.

**Abstract:**

Chitinases are a large and diversified category of enzymes that break down chitin, the world’s second most prevalent polymer after cellulose. GH18 is the most studied family of chitinases, even though chitinolytic enzymes come from a variety of glycosyl hydrolase (GH) families. Most of the distinct GH families, as well as the unique structural and catalytic features of various chitinolytic enzymes, have been thoroughly explored to demonstrate their use in the development of tailor-made chitinases by protein engineering. Although chitin-degrading enzymes may be found in plants and other organisms, such as arthropods, mollusks, protozoans, and nematodes, microbial chitinases are a promising and sustainable option for industrial production. Despite this, the inducible nature, low titer, high production expenses, and susceptibility to severe environments are barriers to upscaling microbial chitinase production. The goal of this study is to address all of the elements that influence microbial fermentation for chitinase production, as well as the purifying procedures for attaining high-quality yield and purity.

## 1. Introduction

Chitin, an insoluble polysaccharide, is the structural component of many organisms, such as mollusks, crustaceans, algae, fungi, and marine invertebrates [1]. Chitin, along with its derivatives, is of high industrial importance and possesses applications in medicine, dermatology, cosmetics, food, and agricultural sectors. Chitinases are enzymes that hydrolyze chitin, and mainly belong to four glycoside hydrolase (GH) families, 18, 19, 23, and 48 [2,3]. In chitin and chitodextrins, chitinases randomly cause the endo-hydrolysis of N-acetyl-β-D-glucosaminide (1→4)-β-linkages [4].

Chitinases convert the chitin polymer into chitooligosaccharides (COS), which are then converted to N-acetyl-D-glucosamine units by the action of chitobiases [5,6]. COS have several important biological activities, such as antimicrobial, antioxidant, anti-inflammatory, angiotensin I-converting enzyme (ACE) inhibiting, immunostimulating, antitumor, and hypocholesterolemic activities, as well as the ability to enhance the absorption of iron and calcium [7]. Further, since chitinous waste is generated in abundance, its sustainable valorization is essential to prevent environmental pollution. In this context, chitin-active enzymes are critical toolboxes for chitin waste management, with simultaneous generation of value-added products [8]. Apart from their myriad of industrial applications, chitinases, as biocontrol agents, are gaining popularity in the agricultural sector, for increasing crop productivity. They offer a safe alternative to toxic pesticides for the protection of crops, due to their unique ability to inhibit pathogenic fungi and insects without disturbing plants, vertebrates, and other components of an ecosystem.

Various groups of organisms, including plants, insects, bacteria, actinomycetes, and fungi, secrete chitinases. Though researchers report a few extremophilic chitinases secreted by fungi and plants, bacteria and archaea are the primary sources of extremophilic chitinases. Extremophilic chitinases can withstand extreme conditions, such as high salt concentrations, and extreme pH and temperature, making them suitable candidates for various industrial processes [9]. Since fermentation conditions and medium composition critically affect chitinase production from extremophilic, as well as mesophilic organisms, the review comprehensively covers the diverse factors impacting the microbial production of chitinase.

In order to grasp an understanding of the catalytic potential of distinct chitin-degrading enzymes, the review also provides vital insights into their widely varied categorization and structural organization. The current study focuses on the microbiological potential for producing cost-effective and robust chitinases, as well as the in depth exploration of their applications in various industries. The possibility of using a recombinant biotechnological tool or immobilization to boost the native catalytic efficacy has also been considered. 

## 2. Classification of Chitin-Degrading Enzymes

**Figure 1:**
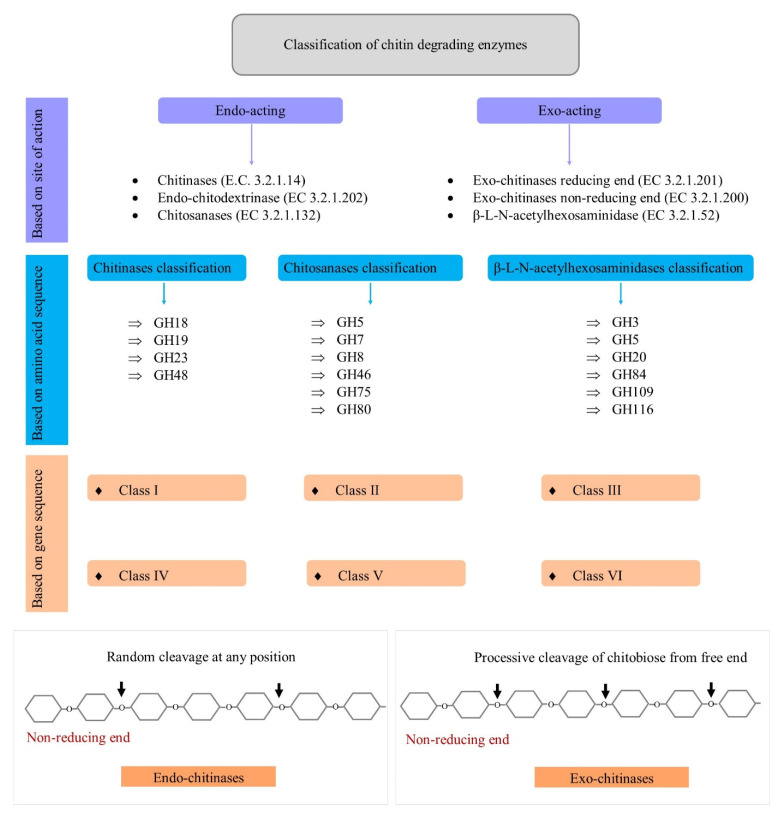
Classification of chitin-degrading enzymes.

The enzyme commission has classified chitinolytic enzymes into different types, namely, chitinases (EC 3.2.1.14), exo-chitinase (reducing end) (EC 3.2.1.201), exo-chitinase (non-reducing end) (3.2.1.200), β-L-N-acetylhexosaminidases (EC 3.2.1.52), endo-chitodextrinases (EC 3.2.1.202), and chitosanases (EC 3.2.1.132) [4,5]. Various schemes of classification, based on different criteria, are subsequently discussed, and are schematically represented in Figure 1.

### 2.1. Site of Action-Based Classification

Chitin-degrading enzymes are categorized into endo- and exo-acting enzymes, based on their site of action. Chitinases (E.C 3.2.1.14), endo-chitodextrinases (EC 3.2.1.202), and chitosanases (EC 3.2.1.132) are endo-acting enzymes. Chitin is cleaved at random positions along the internal chain by chitinases (EC 3.2.1.14), to generate soluble oligomers of N-acetylglucosamine. Another endo-acting enzyme, endo-chitodextrinase (EC 3.2.1.202), is encoded by the endo I gene, and it hydrolyzes chitodextrins to release N, N′-diacetylchitobiose, and small amounts of N, N′, N′′-triacetylchitotriose. An endo-cleaving chitodextrinase (EC 3.2.1.202), characterized from the bacterium *Vibrio furnissii*, participates in the chitin catabolic pathway found in members of *Vibrionaceae*, and, dissimilarly to chitinase (EC 3.2.1.14), it has no activity on chitin [4]. Chitosanases (EC 3.2.1.132) are also endo-acting enzymes that cause endohydrolysis of the β-(1→4)-linkages between D-glucosamine residues in a partly acetylated chitosan [5,10].

Exo-chitinase (reducing end) (EC 3.2.1.201), exo-chitinase (non-reducing end) (3.2.1.200), and β-L-N-acetylhexosaminidases (EC 3.2.1.52) are exo-acting enzymes. Exo-chitinase (reducing end) (EC 3.2.1.201), encoded by the *chiA* gene, acts on the reducing end of chitin and chitodextrins, and causes the hydrolysis of N, N′-diacetylchitobiose. It liberates N, N′-diacetylchitobiose disaccharides by hydrolyzing the second glycosidic (1→4) linkage on the reducing end of chitin and chitodextrin molecules. The *chiB* gene encodes exo-chitinase (non-reducing end) (EC 3.2.1.200), and it generates N′-diacetylchitobiose from the non-reducing end of chitin and chitodextrins. Initially, exochitinases were subcategorized into chitobiosidases (EC 3.2.1.29) and 1,4-β-glucosamidases (EC 3.2.1.30) [4]. However, according to NC-IUBMB (the Nomenclature Committee of the International Union of Biochemistry and Molecular Biology), EC 3.2.1.29 (chitobiosidases) and EC 3.2.1.30 (1,4-β-glucosamidases) have now been deleted and included in EC 3.2.1.52 (β-L-N-acetylhexosaminidase). EC 3.2.1.52 (β-L-N-acetylhexosaminidase) was created in 1972. In contrast, EC 3.2.1.30 (1,4-β-glucosamidases) was created in 1961 and incorporated into EC 3.2.1.52 (β-L-N-acetylhexosaminidase) in 1992, and EC 3.2.1.29 (chitobiosidases) was created in 1961 and incorporated into EC 3.2.1.52 (β-L-N-acetylhexosaminidase) in 1972 [4].

### 2.2. Gene Sequence-Based Classification

Chitinases are classified into six different classes, based on their gene sequence [11,12]. The class of chitinases is determined by various characteristics, such as their isoelectric pH, signal peptide, the sequence at the N-terminal, inducers, and localization of the enzyme.

The chitinases in class I have a cysteine-rich N-terminal. These undergo vacuolar localization, and possess valine or a leucine-rich signal peptide. These are further subdivided into two different classes, based on their acidic or basic nature. Plant chitinases, mostly endo-chitinases, are presented in class I.

Class II chitinases have a sequence similar to class I chitinases, but the cysteine-rich N-terminal is not present in class II chitinases. This class consists of only exo-chitinases, and contains chitinases from plants, fungi, and bacteria.

The sequence of class III chitinases is different from class I or class II chitinases. Class III chitinases have a GH18 catalytic domain with (β/α)_8_-barrel folding. These possess three conserved disulfide bonds, and the folding is somewhat similar to that of bacterial and fungal chitinases, despite the low sequence similarity.

As compared to class I chitinases, the size of class IV chitinases is significantly smaller, but they have similar characteristics, including immunological properties.

The chitinases in class V and class VI are not characterized well enough. Based on the gene sequence, it is reported that the cysteine-rich N-terminal appeared to be lost during evolution, as observed in one example of a class V chitinase, due to less selection pressure. There are two chitin-binding domains in tandem in class V chitinases [11,12].

### 2.3. Amino Acid Sequence-Based Classification

Based on their amino acid sequence, chitin-degrading enzymes are classified into four different GH families in the CAZy database [13], whereas β-L-N-acetylhexosaminidases and chitosanases are classified into six other GH families. Overall, Table 1 enlists the characteristics of chitin-degrading enzymes belonging to different GH families.

#### 2.3.1. GH Family of Chitinases 

According to the CAZy database [3,13], chitinases are mainly grouped into four different GH families, i.e., GH18, GH19, GH23, and GH48. GH18 family chitinases belong to the GH-K clan. The glycosidic bonds of chitin are cleaved by these enzymes with the retaining mechanism of configuration, and their catalytic domain has a (β/α)_8_-barrel 3D structure. Glutamate acts as a proton donor, and the carbonyl oxygen of the C-2 acetamido group of the substrate acts as a nucleophile. This family contains chitinases of class III and V. Chitinases belonging to the GH19 family cleave the glycosidic bonds with an inverting mechanism of configuration, and this family contains chitinases of classes I, II, and IV. The information about proton donors and nucleophiles in the reaction catalyzed by GH19 family chitinases is not known. Chitinases belonging to the GH23 family possess glutamate as a catalytic proton donor. GH48 chitinases cleave the glycosidic bonds with an inverting mechanism of configuration. Their catalytic domain has an (α/α)_6_-barrel 3D structure and belongs to the GH-M domain. Glutamate acts as a proton donor, and the information about catalytic nucleophiles is not known [3,13].

#### 2.3.2. GH Family of β-L-N-acetylhexosaminidases

β-L-N-acetylhexosaminidase (EC 3.2.1.52) enzymes cause the hydrolysis of terminal non-reducing N-acetyl-D-hexosamine residues in N-acetyl-β-D-hexosaminides. In the CAZy database, they are grouped into six different GH families (GH3, GH5, GH20, GH84, GH109, and GH116) [3,13]. 

#### 2.3.3. GH Family of Chitosanases

Chitosanases (EC 3.2.1.132), produced by some bacteria, deacetylate chitin to chitosan, which is finally converted to glucosamine residues. In the CAZy database, these are grouped into six different GH families (GH5, GH7, GH8, GH46, GH75, and GH80) [3,13].

## 3. Structural Organization of Chitinases

Various researchers have demonstrated the 3D structure of different chitinases belonging to the GH18 family. An eight-stranded α/β barrel represents the enzyme core of GH18 family chitinases, where eight strands of parallel β sheets are laid down with an α helix. The eight strands of β sheets bend into a barrel structure, with the helices forming a ring towards the outside. Most of the bacterial chitinases belonging to the GH18 family are characterized by a TIM-barrel (triosephosphate isomerase) fold in the catalytic subunit-containing conserved sequence Tyr-10, Ser-93, Asp-140 [14]. These enzymes degrade chitin with a retention mechanism of configuration. The 3D structure of the chitinase ChiA74 from *Bacillus thuringiensis* was elaborated by Juárez-Hernández et al. [15]. The crystal structure had the following four domains: a chitin-binding domain, fibronectin type III, chitinase insertion domain for barrel insertion, and the catalytic region with an (α/β)_8_-TIM barrel as a core structure. ChiA74 contains 676 amino acids that possess an N-terminal His-tag. The 442 residues form the catalytic region, which has a substrate-binding cleft and a semi-closed tunnel-shaped active site, with both an (α/β) 8-TIM barrel and CID domain. There are three disulfide bonds in the TIM barrel between the cysteine residues, at C78–C100, C140–C146, and C453–C461, and one in the CID domain at C348–C359. The FnIII linker loop (residues 476–484 at the opposite site of the catalytic region) links the FnIII-like domain to the TIM barrel. A calcium ion is present in a highly coordinated form in ChiA74. The CID domain containing 82 residues is present between the seventh beta-strand and the seventh alpha-helix of the (α/β)_8_-TIM barrel. 

A similar structure was reported by Liu et al. [16] for the insect chitinase OfChi-h. Two domains, the fibronectin III domain and the catalytic domain, are connected via a linker of 25 amino acids. These domains were found to interact with each other via a motif consisting of two antiparallel β-strands and one short α-helix. The fibronectin III domain is an immunoglobulin-like β-sandwich domain that comprises eight β-strands, and the catalytic domain is a (β/α)_8_-barrel fold composed of eight β-strands and eight α-helices. A chitinase insertion domain, consisting of five antiparallel β-strands flanked by two α-helices, was also observed in the catalytic domain.

In GH19 family chitinases, 10 α-helical segments and one three-stranded β sheet are present in the secondary structure [17]. GH18 family chitinases catalyze the hydrolytic reaction by substrate-assisted mechanisms, whereas GH19 family chitinases have a high percentage of α-helices and adopt the single-displacement catalytic mechanism [18].

## 4. Sources of Chitinases

### 4.1. Plant Chitinases

Chitinases are expressed during various developmental stages of the plant, especially in response to biotic stress, to protect the plant against phytopathogens. Chitinase genes have been reported in plants such as *Arabidopsis*, *Euphorbia characias*, *Solanum lycopersicum*, and *Oryza sativa*. In *Arabidopsis*, 25 chitinase family genes have been identified, while, in rice, 49 chitinase family genes have been identified so far. Based on the available database, 43 chitinases have been identified in tomatoes [19]. Moreover, the plant genome contains many genes that code for catalytically inactive chitinases, also referred to as chitinase-like proteins (CLPs). These CLPs lack catalytic activity/binding ability due to substitutions in the chitin-binding domain (CBD) [20].

The expression of some proteins increases significantly in plants when the plants are exposed to different pathogens or stresses. These proteins are defined as PR (pathogenesis-related) proteins. These are a component of the plant’s systemic acquired resistance (SAR), and the expression of these PR proteins enhances the plant’s resistance to biotic (pathogens) and abiotic stresses (salinity and heavy metals). In the apple plant, the PR4 group is responsible for chitin recognition and the resistance response. In many plant species, the cell polarity of pathogenic fungi is destroyed by PR4 proteins, by binding at the tip of fungal hyphae [21]. The PR proteins are classified into 17 families, based on molecular mass, isoelectric point, localization, and biological activity [22,23]. These include β-1,3-glucanases, chitinases, thaumatin-like proteins, proteinase inhibitors, endoproteinase, peroxidases, ribonuclease-like proteins, thionins, nonspecific lipid transfer proteins, oxalate oxidase and oxalate oxidase-like proteins, and defensin. Among these 17 families, only four PR families, namely, PR3, PR4, PR8, and PR11, contain chitinases [23].

### 4.2. Chitinases from Insects and Other Organisms

Chitinases have also been reported in arthropods, mollusks, protozoans, nematodes, and coelenterates. Various insects, such as *Drosophila melanogaster*, *Tribolium castaneum*, *Anopheles gambiae*, *Bombyx mori*, *Hyphantria cunea*, *Chironomus tentans*, *Spodoptera litura*, *Choristoneura fumiferana*, *Helicoverpa armigera*, *Aedes aegypti*, *Culex quinquefasciatus*, *Lacanobia oleracea*, *Spodoptera exigua*, *Mamestra brassicae*, *Ostrinia furnacalis*, and *Acyrthosiphon pisum*, are known to produce chitinases [24,25]. In three mosquito species (*Anopheles gambiae*, *Aedes aegypti*, and *Culex quinquefasciatus*), multiple chitinase genes have been identified [24]. The first full-length cDNA of an insect chitinase gene was cloned from *Manduca sexta* [26]. Chitinase genes have also been isolated from different crustaceans, such as the Pacific white shrimp (*Litopenaeus vannamei)*, Chinese mitten crab (*Eriocheir sinensis)*, black tiger shrimp (*Penaeus monodon)*, ridge tail white prawn (*Exopalaemon carinicauda)*, and juvenile kuruma shrimp (*Marsupenaeus japonicus)* [27]. Acidic mammalian chitinase (AMCase) and chitotriosidase (Chit1) are two different types of chitinases that are produced by mammals. Pigs have also been reported to produce chitinases. The expression of chitinases in pig’s stomachs can be induced by the presence of chitin in their diet (insect powder). Kawasaki et al. [28] reported the production of acidic mammalian chitinase in piglets.

### 4.3. Microbial Chitinases

Chitinases are secreted by various microorganisms, such as bacteria, actinomycetes, fungi, and other organisms, as well as plants. Several bacteria (*Bacillus*, *Streptomyces*, *Brevibacillus*, *Serratia*, and *Chromobacterium*) [29] and fungi (*Aspergillus*, *Trichoderma*, *Neurospora*, *Mucor*, *Lycoperdon*, *Metarhizium*, *Beauveria*, and *Lecanicillium*) [30] are known for their chitinase production abilities. Table 2 summarizes the chitinases produced by these microbes, and their major characteristics. 

#### 4.3.1. Bacteria

Chitinolytic bacteria can decompose chitin under both aerobic and anaerobic conditions, and have been isolated from soil, hot springs, compost, and shellfish waste. Several bacteria, including *Alteromonas*, *Aeromonas*, *Bacillus*, *Escherichia*, *Paenibacillus*, *Pseudomonas*, *Serratia*, *Streptomyces*, and *Vibrio*, have been popularly reported for their chitinase activity [5,51,64]. Marine bacteria are also an important source of chitinases, and play an important role in chitin recycling in the aquatic environment; these include, in particular, *Bacillus*, *Vibrio*, *Paenibacillus*, *Pseudoalteromonas*, *Aeromonas*, *Micrococcus*, *Streptomyces*, *Alteromonas*, *Actinomyces*, *Chitiniphilus*, and *Achromobacter* [65]. Fu et al. [66] reported a chitinase of 67 kDa from the marine thermophilic bacterium *Paenicibacillus barengoltzii* CAU904, with an optimum pH and temperature of 3.5 and 60 ℃, respectively. Chitinases from extremophilic bacteria are important, due to their resistance to multiple factors, such as salt, temperature, heavy metals, etc. Thermostable chitinases are ideally suited for industrial purposes, as they can withstand the high-temperature operating conditions, which enable enhanced solubility of hydrophobic compounds and prevent microbial contamination [10]. A hyperthermostable chitinase from *Brevibacillus formosus* BISR-1, with a half-life of more than 5 h at 100 ℃, was isolated by Meena et al. [67]. Li et al. [68] characterized a thermophilic chitinase from the marine bacterium *Microbulbifer* sp. BN3, and the enzyme was stable at a high temperature, with maximum activity at 60 ℃. The thermostability of the chitinase enzyme can also be increased by site-directed mutagenesis and other molecular tools. Engineering of the *Paenibacillus pasadenensis*-derived chitinase PpChi1, by combinational mutagenesis and semi-rational design, improved the optimal temperature to 52.5 ℃, from 45 ℃ [45]. 

#### 4.3.2. Actinomycetes

Actinomycetes are known for their antagonistic activities against various pathogens, due to their ability to produce a wide array of antibiotics. Besides antibiotics, actinomycetes are prolific producers of chitinases. Both mesophilic genera, such as *Streptomyces*, *Saccharothrix*, and *Microbispora*, and thermophilic genera, such as *Thermobifida* and *Streptosporangium*, are reported to produce chitinases [8,10]. A chitinase-producing actinomycete, *Saccharothrix yanglingensis* Hhs.015, was isolated from the roots of cucumber, which produced eight different chitinases [55]. 

#### 4.3.3. Fungi

Fungal chitinases have been reported from various genera, such as *Mucor*, *Aspergillus*, *Penicillium*, *Trichoderma*, *Beauveria*, *Coprinopsis*, *Metarhizium*, *Pistacia*, and *Lactarius*, etc. The fungal cell wall is made of chitin and, therefore, has to protect itself from self-lysis. Due to cell wall protection in healthy hyphae vs. deprotection during a mycoparasitic attack, hyphal aging, and autolysis, it has been hypothesized that the degradation of self and non-self is regulated by substrate accessibility, rather than the speciation of individual chitinases. Fungi guard their cell walls against their hydrolytic enzymes by producing hydrophobic cell wall proteins, such as QID74 and carbohydrate-binding proteins. Carbohydrate-binding proteins bind to short oligosaccharides and chitin, and, therefore, mask them from degradation [69]. Rosado et al. [70] reported the role of the cell wall protein QID74, produced from *Trichoderma harzianum*, in cell protection and adherence to hydrophobic surfaces. The cell wall becomes more sensitive towards lytic enzymes in the absence of the QID74 protein. Repetitive peptide motifs in hydrophobic cell wall proteins also increase the molecular rigidity and hydrophobicity of cell walls, which helps in cell wall protection. 

The molecular weight of fungal chitinases (35–50 kDa) is generally comparable to bacteria (20–60 kDa). In general, the optimum pH and temperature of fungal chitinases range from 4.0 to 8.0 and 40 to 50 °C, respectively [5,30]. Fungal chitinases exhibit anti-fungal activities against many plant pathogenic fungi, such as *Aspergillus flavus*, *Botrytis cinerea*, *Rhizoctonia solani*, *Aspergillus niger*, *Aspergillus oryzae*, *Penicillum oxysporium*, *Candida albicans*, *Fusarium solani*, and *Sclerotinia sclerotiorum* [59,63,71]. 

Generally, the inherent yield potential of wild organisms is low, which can be improved by the expression of the chitinase gene in a heterologous host, for the production of this enzyme in abundance. Chitinase genes from bacteria, actinobacteria, and fungi have been cloned and expressed in different expression hosts, such as *E. coli* BL21, *Pichia pastoris*, etc. [56,63]. Chitinase yields in heterologous hosts generally increase to a much higher titer (~22.6-fold) as compared to wild organisms. Nonetheless, chitinases from both wild and recombinant microorganisms are commercially available. Many chitinase formulations from wild strains of *Aspergillus niger*, *Streptomyces griseus*, and *Trichoderma viride* are commercially available. Recombinant chitinases are also commercially available, and include chitinase 18A from *Clostridium thermocellum* (activity—25 U/mg), chitinase 18A from *Bacillus cereus* (concentration—1 mg/mL), chitinase 18A from *Bacillus licheniformis* (concentration—1 mg/mL), and chitinase 18A from *Clostridium thermocellum* (activity—25 U/mg). These recombinant chitinases are produced at a large scale using *E. coli* (https://www.creative-enzymes.com/). Merck (Sigma-Aldrich), Megazyme, and Prospec Protein Specialists are the major commercial producers of chitinase. Prospec Protein Specialists manufacture a recombinant chitinase under the product name Chitinase Protein from *Clostridium paraputrificum*, expressed in *E. coli* (https://www.prospecbio.com/, 10 April 2021). Table 3 enlists the commercially available microbial chitinases.

## 5. Microbial Fermentation for Chitinase Production

Both submerged fermentation (SmF) and solid-state fermentation (SSF) have been effectively used to produce chitinase from bacteria or fungi [72]. Bacteria are preferred for chitinase production, as compared to fungi, because of their high volumetric productivity [73]. For large-scale chitinase production, the substrate chitin is pretreated to increase its accessibility to chitinase, which helps to significantly improve the yields. Different techniques, such as ultra-sonication, acid treatment, and steam explosion, are used for the pretreatment of chitin. Lactic acid is also used to process chitin, which is a more environmentally favorable approach than hydrolysis using hydrochloric acid. Chitin processed using lactic acid has a higher potential to induce chitinase production when compared to colloidal chitin.

### 5.1. Submerged Fermentation (SmF) for Chitinase Production

SmF is the most widely used method for chitinase production from different microbes, such as *Streptomyces pratensis* strain KLSL55 [74], *Trichoderma viride* [75], *Aspergillus niveus* [76], *Beauveria bassiana* [77], and *Bacillus cereus* GS02 [78]. The fermentation may be operated in batch, fed-batch, or continuous mode, depending on the type of substrate and microorganism being used. SmF has several advantages, including enhanced oxygen delivery, increased mass transfer, and better process control. Different bioreactors, such as an airlift bioreactor, an airlift with a net draft tube reactor, a stirred-tank bioreactor, and a bubble column reactor, have been used for chitinase production [72].

### 5.2. Solid-State Fermentation (SSF) for Chitinase Production

Nutrient-rich waste materials, such as bagasse, paper pulp, and bran, can be used as substrates in SSF, for producing different metabolites, including chitinases. Chitin, being a solid material, can be directly used as the substrate for SSF, which potentially reduces the cost of the enzyme [72]. Chitinase production using SSF has been studied in various microorganisms, such as *Trichoderma koningiopsis* UFSMQ40 [79], *Chromobacterium violaceum* [80], *Metarhizium anisopliae* [81], and *Bacillus thuringiensis* R 176 [82]. The use of SSF for large-scale chitinase production is generally limited to laboratory scale, despite the low cost and high titer [83].

### 5.3. Factors Affecting Chitinase Production by Fermentation

Numerous physico-chemical factors, including the substrate, pH, agitation speed, fermentation period, inoculum size, carbon source, nitrogen source, metal ions, and temperature, affect the growth of chitinase-producing microorganisms and the subsequent titer of chitinase. These factors vary with the type of microbe used, and also with the method used for chitinase production. The effect of these factors on chitinase production is discussed below.

#### 5.3.1. Substrate Used

Chitinase production is induced by the presence of (1,4)-glycosidic bonds in the substrate. Though various substrates, such as powdery chitin, colloidal chitin, chitosan, crab shell, shrimp powder, demineralized crab, wheat bran, sugarcane bagasse, and cellobiose, are used for the production of chitinase [64,79,84,85], colloidal chitin is the substrate of choice at the commercial scale, due to its higher productivity [64,86]. Colloidal chitin, chitin flakes, and other chitinous substrates, such as dimeric N-acetylglucosamine, are good inducers of chitinase. Glucose and xylose are also known to induce chitinase production in *Streptomyces* sp. K5 [84], and chitobiose was reported to be a strong inducer of chitinase in *Trichoderma asperellum* T4 [87].

#### 5.3.2. Temperature and pH

Both temperature and pH are important factors that influence chitinase production. The fermentation temperature changes with the microorganism used. Most of the commercial strains used are mesophilic (*Bacillus* sp., *Streptomyces* sp., *Aspergillus* sp., and *Trichoderma* sp.), but a few thermophiles are also used at the commercial scale. The optimum pH for maximum chitinase production by various microorganisms generally varies between 6.0 and 8.0 [2,74,86,88].

#### 5.3.3. Agitation Speed

As compared to static conditions, a good titer of chitinase is obtained at a higher rpm (revolutions per minute). However, when agitation is increased beyond a certain value, it causes cell sufferance and, consequently, low enzyme production, due to shear stress. Shear stress was also reported to partially inactivate the enzyme [89]. An airlift reactor with a net draft tube fermenter is generally used to improve agitation at the commercial scale.

#### 5.3.4. Fermentation Period

For the production of chitinase, the fermentation period varies with the organism, substrate used, and other process parameters. The incubation time varies from 24 to 72 h in bacteria [73,74,86] and 48 to 96 h in fungi under SmF [79,90]. It was also reported that *Bacillus* and *Paenibacillus* require a shorter cultivation period (2–3 days) for chitinase production than *Streptomyces thermocarboxydus* TKU045, which takes 5 days [91].

#### 5.3.5. Inoculum Size

Microbial growth and growth-related parameters are improved by increasing the inoculum size up to a certain level (1–5%, *v*/*v*), and a reduction in microbial activity is observed when the inoculum size is further increased, due to nutritional limitation. Gupta, Kumar, Laksh, and Rana [73] reported a maximum enzyme production of 0.53 IU with 1% of a 24 h starter culture of *Bacillus* sp. For *Streptomyces pratensis* strain KLSL55, production increased with inoculum size, and the highest titer of 141.20 IU was obtained with 1.25% inoculum (1 × 10^8^ spores/mL) [74].

#### 5.3.6. Carbon and Nitrogen Source

Yeast extract, malt extract, casein, ammonium sulfate, colloidal chitin, and chitin flakes are commonly used nitrogen sources for higher production [73]. The maximum chitinase production by *Bacillus* sp. R2 was observed with yeast extract as a nitrogen source [85], while, for *Streptomyces macrosporus* M1, the chitinase production was the highest with KNO_3_ [92]. Swollen chitin, sucrose, and cellulose improved the chitinase production from *Chitinophaga* sp. S167 [2], whereas starch increased the chitinase production from *Paenibacillus* sp. [51], when provided as a carbon source.

#### 5.3.7. Metal Ions

The addition of Mn^2+^, Ca^2+^, and Co^2+^ improved the yield of chitinase, while Cu^2+^, Zn^2+^, Mg^2+^, and Fe^2+^ decreased the yield in bacterial fermentation [49,74,92]. Stimulation of the enzymatic activity in the presence of metal ions may be due to a change in electrostatic bonding. These metal ions may act as a binding link between the enzyme and the substrate. Ca^2+^ helps extracellular enzymes to withstand high temperatures, and is a well-known stimulator. The residues of aspartic and glutamic acid in chitinases bind to certain divalent cations, such as Zn^2+^ and Hg^2+^, which can inhibit chitinases [93].

### 5.4. Purification of Chitinases

Enzymes are needed in purified form to obtain information about their amino acid sequence, biochemical functions, and evolutionary relationships between proteins in diverse organisms [94]. For the purification of chitinase, the culture filtrate is generally precipitated using the ammonium sulfate precipitation method, followed by the use of various chromatography techniques for further purification (Table 4). Different researchers have obtained 4.3–14.4-fold purification with a recovery yield of 18.4–2.6%. The molecular weight of the purified chitinases varies from 20 to 120 kDa, with a pI range from 4.5 to 8.5.

Further, the efficiency and stability of purified free chitinases are generally less, which can be improved by immobilization [97]. Enzyme immobilization is used to achieve stable, reusable, and more active enzymes by the simple act of fixing an enzyme on a support surface [98]. Various types of supports are used for immobilization, and, amidst them, magnetic nanoparticles (MNPs) of 10 to 20 nm are highly favored, due to their non-toxicity, large surface area and surface: volume ratio, and the simplicity in separating the biocatalyst after use. A chitinase immobilized on MNPs can be recovered easily, and can be used multiple times without losing much activity [99]. The immobilized enzyme is reported to be more active for COS production as compared to the free enzyme [97]. Different activators and cross-linking agents, such as 3-aminopropyl triethoxylsilane (APTES), cyanogen bromide, etc., are popularly used for the preparation of immobilized enzymes. Glutaraldehyde cross-linked chitosan beads were used to immobilize a recombinant, thermostable fungal chitinase from *Thermomyces lanuginosus*, which further increased the stability of the immobilized chitinase, as compared to the free enzyme [100]. Similarly, the stability and catalytic activity of the purified recombinant Chit36 enzyme were improved by immobilization on Ca^2+^cross-linked alginate beads and Ca^2+^ cross-linked alginate–sepiolite nanocomposite beads [101]. Though the immobilization of chitinases looks promising, more research is needed on non-toxic cross-linkers, especially for food-related applications.

### 5.5. Applications of Chitinases

Chitinases have a wide array of applications in various fields, including medical, industrial, and agricultural, which are elaborately discussed below.

#### 5.5.1. Biocontrol Agent

Several pathogens, such as *Fusarium* sp., *Botrytis cinerea*, *Sclerotinia sclerotiorum*, and *Rhizoctonia solani*, possess chitin as the main cell wall constituent. Insects also contain chitin in their exoskeleton, which helps them to survive in adverse conditions. Chitinases can be used against both phytopathogenic fungi and insect pests. Chitinase from *Penicillium ochrochloron* has shown biocontrol activity against *H. armigera*, by increasing larval and pupal mortality, and reducing pupation [102]. The chitinase gene from *Xenorhabdus nematophila* was cloned in *E. coli* (DH5α) using a pGEMT easy vector, and expressed in *E. coli* BL-21 (DE3) using a pET-28b vector. This heterologous recombinant chitinase showed biocontrol activity against the larvae of *H. armigera*. The LD_50_ value was 20 µg/g, which caused 51.5% larval mortality on day 3 [47]. Further, chitinase production is also induced by the attack of phytopathogens in plant seeds, stems, tuber, and flowers. Some elicitors (such as COS) or growth regulators (ethylene) induce the production of chitinases, which act as PR proteins in plant self-defense [103].

#### 5.5.2. Single-Cell Protein (SCP) Production

Single-cell proteins are used as food supplements, and are generated from single-celled microorganisms. Chitin-containing waste can be used as a substrate (shellfish chitin, shrimp cell waste, prawn cell waste, etc.) by chitin-utilizing microorganisms to produce SCP. Chitinases hydrolyze the chitin present in the waste products and release simpler products, which can be further used to grow yeast and other microbes to generate SCP. Various researchers have demonstrated the use of chitinase to produce SCP [104,105]. Patil and Jadhav [104] hydrolyzed shellfish chitin using chitinase from *Penicillium ochrochloron* to generate N-acetyl-D-glucosamine, which was used as the substrate for SCP production using *Yarrowia lipolytica* NCIM 3450.

#### 5.5.3. Protoplast Isolation

Protoplasts are crucial for physiological and genetic studies on fungi [106]. Fungal protoplasts have been extensively used for the preparation of cell-free extracts and fungal organelles, for studying enzyme synthesis and secretion, cell wall synthesis and characterization, and genetic recombination and transformation. Chitinases are widely used to prepare fungal protoplasts of many fungi, including *T. reesei*, *P. florida*, *A. bisporus*, and *A. niger* [107].

#### 5.5.4. Biomedical Applications

Chitinases also play important roles in the regulation of humoral and cellular immune responses. Allergens and helminths are known to induce the production of acidic mammalian chitinase (AMCase), which functions as a critical initiator of protective type 2 responses against intestinal nematodes. In an experiment conducted on AMCase-deficient mice, it was observed that when the mice were infected with the chitin-containing gastrointestinal nematodes *Nippostrongylus brasiliensis* and *Heligmosomoides polygyrus bakeri*, there was no type 2 immune response present [108]. Similarly, the chitinase produced by *Lactobacillus rhamnosus* GG inhibited the morphogenesis of the opportunist pathogen *Candida albicans* [109].

#### 5.5.5. Chitooligosaccharide (COS) Production

Chitinases produce COS, which have immune-stimulant, anti-inflammatory, antibacterial, antioxidant, and prebiotic properties [91]. Chitin and COS are gaining interest due to their biocompatibility, non-toxicity, and biodegradability, which make them suitable for various applications. COS produced by the chitinase CsChiE from *Chitiniphilus shinanonensis* SAY3 efficiently hydrolyzed unmilled crab shell chitin waste (chitin flakes), and crystalline α-, β- and colloidal chitin. The predominant hydrolysis product was chitobiose, which is used as a cosmetic ingredient, dietary supplement, and in osteoarthritis therapeutics [48]. The chitinase PbChi70, from the thermophilic marine bacterium *Paenibacillus barengoltzii*, can efficiently produce N-acetyl chitobiose (GlcNAc)_2_ [35]. The hydrolysis of colloidal chitin by *Streptomyces thermocarboxydus* chitinase produced chitin oligomers with multiple degrees of polymerization (DP). The chitin oligomers with low DP exhibited enhanced 2, 2-diphenyl-1-picrylhydrazyl radical scavenging capability and promoted the growth of probiotic *Lactobacillus lactis* [91]. Pan, Li, Lv, Du, and Liu [39] increased the chitinase production from *Bacillus* sp. DAU101 for the enzymatic production of COS using molecular engineering. The expression level of chitinase increased from 35.54 U/mL to 51.67 U/mL by optimizing the ribosome binding sites (RBSs) with spacer sequences, and combining molecular docking technology with site-directed mutagenesis.

#### 5.5.6. Waste Management

Waste from the sea, containing crab, lobster, and shrimp shells, is a challenging problem that requires massive landfills for disposal. Globally, approximately six to eight million tons of chitinous waste is produced annually. Chitinous waste can be degraded using physical and chemical methods, but these methods are expensive and not environmentally friendly. Chitinases can be an alternative eco-friendly approach for chitinous waste management. The application of chitinases is known to enhance the degradation rate of waste, and is being utilized for the management of chitinous waste globally [110]. An overexpressed chitinase from *Bacillus subtilis* degraded pretreated crystalline chitin substrates (such as α-chitin, β-chitin, and crab shells) more efficiently than SmChiA chitinase from *Serratia marcescens* and the commercial chitinase preparation from *Streptomyces griseus* [111].

## 6. Conclusions and Future Prospects

Chitinase, a commercially significant enzyme produced by bacteria, fungi, and insects, has a wide range of applications, including waste management, the production of SCP and COS, protoplast generation, biocontrol, etc. Its use as a biocontrol agent in agriculture has significant ramifications as a non-toxic alternative to chemical pesticides and insecticides. At the industrial scale, SmF is the technique of choice for chitinase production, but greater productivities can be obtained using SSF, which requires additional research, especially on heat transfer and downstream processing aspects, before commercial economic acceptability. Although immobilized chitinases offer the potential to reduce the cost of products such as COS, finding non-toxic cross-linkers and activators remains a major difficulty in developing immobilized enzyme formulations for food applications. Because of the numerous applications of chitinases, their demand is expected to rise in the near future.

## Figures and Tables

**Table 1:** Characteristics of chitin-degrading enzymes belonging to different GH families.

GH Family	Clan	Mechanism	Catalytic Domain 3D Structure	Catalytic Nucleophile/Base	Catalytic Proton Donor	Enzyme Name
GH3	-	Retaining	-	Aspartate	Glutamate (for hydrolases)/Histidine (for phosphorylases)	β-L-N-acetylhexosaminidase
GH5	GH-A	Retaining	(β/α)_8_-barrel	Glutamate	Glutamate	Chitosanase, β-L-N-acetylhexosaminidase
GH7	GH-B	Retaining	β-jelly roll	Glutamate	Glutamate	Chitosanase
GH8	GH-M	Inverting	(α/α)_6_	Aspartate	Glutamate	Chitosanase
GH18	GH-K	Retaining	(β/α)_8_-barrel	Carbonyl oxygen of C-2 acetamido group of the substrate	Glutamate	Chitinase
GH19	-	Inverting	-	-	-	Chitinase
GH20	GH-K	Retaining	(β/α)_8_	Carbonyl oxygen of C-2 acetamido group of the substrate	Glutamate	β-L-N-acetylhexosaminidase
GH23	-	-	-	-	Glutamate	Chitinase
GH46	-	Inverting	-	Probably aspartate	Probably glutamate	Chitosanase
GH48	GH-M	Inverting	(α/α)_6_-barrel	-	Glutamate	Chitinase
GH75	-	Inverting	-	Probably aspartate	Probably glutamate	Chitosanase
GH80	GH-I	Inverting	α+β	-	-	Chitosanase
GH84	-	Retaining	(β/α)_8_-barrel	Carbonyl oxygen of C-2 acetamido group of the substrate	Aspartate	β-L-N-acetylhexosaminidase
GH109	-	Retaining	-	Not applicable	None	β-L-N-acetylhexosaminidase
GH116	GH-O	Retaining	(α/α)_6_-barrel	Glutamate	Aspartate	β-L-N-acetylhexosaminidase

Source of information: CAZy database [3,13].

**Table 2:** Chitinases from different microbial sources and their characteristics.

S.No.	Organism Name	Class of Enzyme	Molecular Weight (kDa)	Optimum pH/Temperature (°C)	Reference
Bacteria					
1.	*Pseudoalteromonas* sp. DL-6	GH18	113.5	8.0/20	Wang et al. [31]
2.	*Pseudoalteromonas* sp. DC14,	-	65	9.0/40	Makhdoumi et al. [32]
3.	*Chitinibacter* sp. GC72	-	65	6.8/40	Gao et al. [33]
4.	*Bacillus licheniformis* LHH100	-	65	4.0/75	Laribi-Habchi et al. [34]
5.	*Paenibacillus barengoltzii*	GH18	70	5.5/55	Yang et al. [35]
6.	*Paenibacillus elgii* HOA73	-	68	7.0/50	Kim et al. [36]
7.	*Hydrogenophilus hirschii*	-	59	5.0/85	Bouacem et al. [37]
8.	*Microbulbifer thermotolerans* DAU221	GH18	60	4.6/55	Lee et al. [38]
9.	*Bacillus subtilis* WB600	GH18	62	5.0/60	Pan et al. [39]
10.	*Corallococcus* sp. EGB	GH18	52.9	6.0/50	Li et al. [40]
11.	*Pseudomonas*	-	-	4.5/35	Liu et al. [41]
12.	*Paenibacillus timonensis* LK-DZ15	GH18	70	4.5/80	Yahiaoui et al. [42]
13.	*Chromobacterium violaceum*	GH18	46	5.0/60	Sousa et al. [43]
14.	*Paenibacillus pasadenensis* CS0611	GH18	69	5.0/50	Guo et al. [44] and Xu et al. [45]
15.	*Chitiniphilus shinanonensis*	GH18	45.3	6.0/50	Bhuvanachandra and Podile [46]
16.	*Xenorhabdus nematophila*	GH18	76	-	Mahmood et al. [47]
17.	*Chitiniphilus shinanonensis*	GH18	58.87	7.0/50	Rani et al. [48]
18.	*Serratia marcescens*	-	55.6	6.0/55	Li et al. [49]
19.	*Bacillus licheniformis* J24	GH18	-	7.0/70	Essghaier et al. [50]
20.	*Paenibacillus* sp.	-	30	4.5/50	Du et al. [51]
Actinomycetes					
1.	*Streptomyces* sp.	-	40	2&6/50	Karthik et al. [52]
2.	*Microbispora* sp. V2	-	35	3.0/60	Nawani et al. [53]
3.	*Thermobifida fusca*	GH18	46.3	6.0–8.0/40–45	Gaber et al. [54]
4.	*Saccharothrix yanglingensis* Hhs.015	-	77.9	7.0/49	Lu et al. [55]
5.	*Streptomyces albolongus* ATCC 27414	GH18	47	5.0/55	Gao et al. [56]
6.	*Streptomyces chilikensis* RC1830	-	10.5	7.0/60	Ray et al. [57]
7.	*Streptomyces alfalfae*	GH19	29	8.0/45	Lv et al. [58]
Fungi					
1.	*Aspergillus terreus*	-	60	5.6/50	Farag et al. [59]
2.	*Humicola grisea*	-	50	3.0/70	Kumar et al. [60]
3.	*Myceliophthora thermophila* C1	-	43	6.0/55	Krolicka et al. [61]
4.	*Aspergillus griseoaurantiacus* KX010988	-	130	4.5/40	Shehata et al. [62]
5.	*Trichoderma harzianum* GIM 3.442	GH18	45	6.0/45	Deng et al. [63]

**Table 3:** Commercially available microbial chitinases.

Trade Name and Producing Firm	Producing Organism	Formulation Type (Solid, Liquid)	Activity (U/g)
Chitinase from *Aspergillus niger* (food grade)/Creative Enzymes^®^	*Aspergillus niger*	Light-brown powdered form	200 *
Native *Streptomyces griseus* Chitinase/Creative Enzymes^®^	*Streptomyces griseus*	Lyophilized powder (essentially salt-free)	>200 *
Native *Trichoderma viride* Chitinase/Creative Enzymes^®^	*Trichoderma viride*	Lyophilized powder form	>600 *
Chitinase (*Clostridium thermocellum*)/Megazyme	*Clostridium thermocellum*	Solid	300 **
Chitinase from *Streptomyces griseus*/Merck (Sigma-Aldrich)	*Streptomyces griseus*	Solid	≥200 ***
Chitinase from *Streptomyces griseus*/Merck (Sigma-Aldrich)	*Streptomyces griseus*	Solid	≥1000 ***
Chitinase from *Trichoderma viride*/Merck (Sigma-Aldrich)	*Trichoderma viride*	Lyophilized powder form	≥600 ***

Information adopted from the following three different websites: * https://www.creative-enzymes.com/, ** https://www.megazyme.com/, *** https://www.sigmaaldrich.com/india.html.

**Table 4:** Different techniques used for the purification of chitinases and their characteristics.

Organism Name	Purification Technique	Molecular Weight (kDa)	Specific Activity (U/mg)	Optimum pH/Temperature (°C)	Reference
*Paenicibacillus barengoltzii*	Ammonium sulfate precipitation (20–40%), ion-exchange chromatography	67	12.4	3.5/60	Fu, Yan, Wang, Yang and Jiang [66]
*Aspergillus niveus*	Ammonium sulfate precipitation, Sephadex G-100 gel filtration chromatography	44	13.3	5.0/65	Alves, de Oliveira Ornela, de Oliveira, Jorge and Guimarães [76]
*Aspergillus griseoaurantiacus*	Ammonium sulfate precipitation, DEAE-cellulose column chromatography, Sephacryl S-300 column chromatography	130	93.75	4.5/40	Shehata, Abd El Aty, Darwish, Abdel Wahab and Mostafa [62]
*Bacillus altitudinis* KA15	Ammonium sulfate precipitation (30 and 60%), Sephacryl S-200 high-resolution size exclusion chromatography, high-performance ion-exchange chromatography (IEX)	43	120,000	4.0/85	Asmani et al. [95]
*Myxococcus fulvus* UM01	Ni-NTA affinity chromatography	26.99	-	7.0/35	Shahbaz and Yu [64]
*Paenibacillus* sp.	Ammonium sulfate precipitation (60–80%), DEAE-IEC, and gel chromatography	30	0.85	4.5/50	Du, Duan, Miao, Zhai and Cao [51]
*Shewanella inventionis* HE3	Ammonium sulfate precipitation (20–80%), gel filtration chromatography	40	41,000	4.0/70	Laribi-Habchi et al. [96]

## Data Availability

Data sharing not applicable.
